# Physics-Aware Spatiotemporal Consistency for Transferable Defense of Autonomous Driving Perception

**DOI:** 10.3390/s26030835

**Published:** 2026-01-27

**Authors:** Yang Liu, Zishan Nie, Tong Yu, Minghui Chen, Zhiheng Yao, Jieke Lu, Linya Peng, Fuming Fan

**Affiliations:** 1 Hubei Key Laboratory of Internet of Intelligence, School of Electronic Information and Communications, Huazhong University of Science and Technology, Wuhan 430074, China; u201313659@alumni.hust.edu.cn (Y.L.); niezs@hust.edu.cn (Z.N.); mhchen212@hust.edu.cn (M.C.); zhihengyao@hust.edu.cn (Z.Y.); lu_jk0018.sy@gx.csg.cn (J.L.); 2 School of Computer Science, Northeast Electric Power University, Jilin 132000, China; 1202500129@neepu.edu.cn; 3 Electric Power Research Institute of Guangxi Power Grid Co., Ltd., Nanning 530000, China; 4 Faculty of Artificial Intelligence in Education, Central China Normal University, Wuhan 430079, China; penglinya@mails.ccnu.edu.cn

**Keywords:** autonomous driving perception, physical adversarial attacks, adversarial robustness, transferability, spatiotemporal consistency

## Abstract

Autonomous driving perception systems are vulnerable to physical adversarial attacks. Existing defenses largely adopt loosely coupled architectures where visual and kinematic cues are processed in isolation, thus failing to exploit physical spatiotemporal consistency as a structural prior and often struggling to balance adversarial robustness, transferability, accuracy, and efficiency under realistic attacks. We propose a physics-aware trajectory–appearance consistency defense that detects and corrects spatiotemporal inconsistencies by tightly coupling visual semantics with physical dynamics. The module combines a dual-stream spatiotemporal encoder with endogenous feature orchestration and a frequency-domain kinematic embedding, turning tracking artifacts that are usually discarded as noise into discriminative cues. These inconsistencies are quantified by a Trajectory–Appearance Mutual Exclusion (TAME) energy, which supports a physics-aware switching rule to override flawed visual predictions. Operating on detector backbone features, outputs, and tracking states, the defense can be attached as a plug-in module behind diverse object detectors. Experiments on nuScenes, KITTI, and BDD100K show that the proposed defense substantially improves robustness against diverse categories of attacks: on nuScenes, it improves Correction Accuracy (CA) from 86.5% to 92.1% while reducing the computational overhead from 42 ms to 19 ms. Furthermore, the proposed defense maintains over 71.0% CA when transferred to unseen detectors and sustaining 72.4% CA under adaptive attackers.

## 1. Introduction

Autonomous driving is transforming transportation through enhanced safety and operational efficiency [1,2]. Modern perception stacks rely heavily on Deep Neural Networks (DNNs), which provide strong visual recognition but also introduce critical vulnerabilities [3,4,5]. Physical adversarial attacks inject real-world perturbations, such as patches or projected patterns, to mislead perception without accessing internal sensor data [6,7,8,9]. These attacks are stealthy and low-cost. They can cause dangerous misclassifications and missed detections, posing severe safety risks for intelligent transportation systems.

Existing defenses remain difficult to deploy in real driving systems. Certified defenses offer provable guarantees but their computational cost scales poorly with high-resolution, multi-sensor inputs, making real-time deployment challenging [10,11,12]. Input purification methods reconstruct or denoise sensor data, yet often distort semantics and incur high false positive rates in benign scenes [13,14,15]. More recent work exploits spatiotemporal consistency between appearance and motion [16,17,18] but typically in a loosely coupled manner, where visual and kinematic cues are processed in separate branches and only compared at score level. As a result, physical consistency is not used as a structural prior, and these defenses still struggle to jointly achieve robustness, transferability, accuracy, and real-time efficiency under realistic, adaptive physical attacks.

We argue that physical trajectories should not be treated as an external verifier, but as an internal organizer of visual representations. Building on this view, we propose a physics-aware trajectory–appearance consistency defense that uses physical motion as a structural prior to audit and correct visual predictions. Our design is based on a simple but important observation: physical adversarial attacks inevitably induce a trajectory–appearance inconsistency [16]. An attacker can make an object look like a car, but cannot fully control its inertial trend, high-frequency detection jitter, or long-term dynamics [19,20]. Genuine objects show stable alignment between how they look and how they move; adversarial objects exhibit a semantic gap, often accompanied by abnormal jitter and unstable tracks. To instantiate this idea, we employ a physics-aware, dual-stream spatiotemporal encoder with endogenous feature orchestration that consumes detector backbone features together with detection boxes, labels, and tracked trajectories as input. Motion is decomposed into low-frequency inertial trends and high-frequency jitter in the frequency domain, producing compact kinematic embeddings. These embeddings then drive the orchestration mechanism: frequency-guided queries probe the visual stream, measure trajectory–appearance discrepancy, and modulate visual features accordingly. The resulting inconsistency is quantified by a Trajectory–Appearance Mutual Exclusion (TAME) energy, which serves as a differentiable measure of physical–visual conflict. We instantiate the encoder with a lightweight Transformer for temporal modeling, but treat it as a generic spatiotemporal processor rather than an architectural novelty, and the defense is calibrated once on a source detector and then reused across different perception stacks.

**Figure 1:**
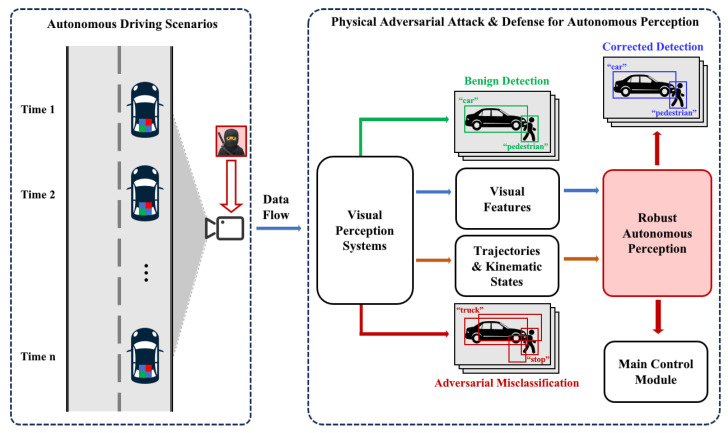
Pipeline of physical adversarial attacks and our defense on autonomous driving perception.

On top of this pipeline, TAME energy enables a transferable physical defense module. Because the module interacts with the perception stack only through backbone features, detector outputs, and tracking states, it can be attached as a plug-in safety layer behind heterogeneous object detectors without modifying their weights or retraining the defense. The combination of frequency-domain kinematic embedding, endogenous feature orchestration, and TAME inconsistency reasoning allows the module to generalize across attack types, including adaptive attacks, as well as across datasets and detector architectures. The overall defense pipeline is illustrated in Figure 1.

The main contributions of this paper are summarized as follows:

• **Dual-stream spatiotemporal encoder with frequency-domain kinematic embedding.** We design a dual-stream spatiotemporal encoder that jointly models visual and kinematic streams. Motion is decomposed into low-frequency inertial trends and high-frequency jitter in the frequency domain, turning tracking artifacts that are often treated as noise into informative cues for trajectory–appearance consistency.
• **Endogenous feature orchestration with TAME inconsistency head.** On top of this encoder, we introduce an endogenous, frequency-guided feature orchestration module that uses kinematic queries to reorganize visual features along the trajectory–appearance consistency manifold. We further define the TAME energy as a differentiable measure of physical–visual conflict, which provides a unified inconsistency head for both attack detection and label correction when visual predictions are compromised.
• **Transferable physical defense module.** We package the encoder, orchestration, and TAME head into a plug-in safety module that can be attached behind heterogeneous object detectors by reusing their backbone features, outputs, and tracking states, without modifying detector weights or retraining the defense. Experiments across multiple datasets, detectors, and both patch-based and projection-based attacks show strong robustness and clear cross-detector/cross-dataset transferability. We further demonstrate that the module maintains nontrivial protection under adaptive attacks such as trajectory smoothing and joint optimization, highlighting the practicality of frequency-guided, physics-aware consistency defense.

## 2. Related Work

### 2.1. Visual Perception for Autonomous Driving

The visual perception stack is at the core of an autonomous vehicle’s ability to interpret its surroundings [1]. It is responsible for real-time analysis of road conditions and directly affects driving safety. Object detection algorithms based on Convolutional Neural Networks (CNNs) remain the dominant approach [21], and recent lightweight architectures can meet the stringent real-time requirements of autonomous driving [22,23,24]. Despite their strong performance in benign scenarios, these models are highly vulnerable to adversarial perturbations. Small but carefully crafted changes to the input can lead to severe misclassifications or complete target loss [25,26,27].

### 2.2. Physical Adversarial Attacks on Autonomous Driving Perception

Adversarial examples are inputs with imperceptible perturbations that cause DNNs to output incorrect predictions [28,29]. Early work mainly focused on digital-domain attacks such as the Fast Gradient Sign Method (FGSM) [30] and Projected Gradient Descent (PGD) [31], where gradient-based perturbations are generated to cross decision boundaries. However, these attacks assume full access to input pixels, which is often unrealistic for deployed autonomous systems. Physical adversarial attacks, by contrast, require perturbations that are feasible and robust in the real world [8,27]. Attackers must contend with illumination changes, viewpoint variations, and sensor noise, and therefore often optimize perturbations under constraints such as Non-Printability Score (NPS) and Total Variation (TV) [32,33].

Adversarial patches are a common vehicle for physical attacks. Robust Physical Perturbations (RP2) generate robust perturbations on road signs that mislead detectors over a wide range of distances and viewing angles [8]. Other methods, such as PatchAttack for vehicles and CAPatch for image captioning, demonstrate the versatility of patch-based attacks across tasks and domains [28,34]. In addition, optical attacks such as Short-Lived Adversarial Perturbation (SLAP) project transient patterns onto object surfaces using a projector [35], enabling non-contact, hard-to-trace attacks that pose serious challenges to visual perception systems.

### 2.3. Physical Adversarial Defenses for Autonomous Driving

Defense strategies against physical attacks can be broadly grouped into three categories. Certified defenses offer provable robustness guarantees through mathematical analysis. For example, Certified Interval Bound Propagation (CertIBP) [10] uses interval bound propagation to bound input perturbations, and PatchGuard [11] constrains localized corruptions via small receptive fields and feature masking. Despite their theoretical rigor, these methods are computationally expensive and scale poorly to high-resolution inputs and multi-sensor settings, limiting their applicability in real-time autonomous driving [36]. Input purification methods aim to remove perturbations at the sensor level. Approaches such as Jujutsu [14] and Diffusion Purification (DiffPure) [13] reconstruct images using classical filters or diffusion models. While they can suppress high-frequency noise, they also tend to erase fine semantic details (e.g., small or distant objects), leading to performance degradation and high false positive rates in benign driving conditions. Spatiotemporal consistency-based defenses leverage temporal information or physical cues to detect anomalies [16,17,18]. PercepGuard [16], for instance, monitors object trajectories to flag predictions that are inconsistent with motion patterns, while PhySense integrates additional physical attributes and relational cues [17,37].

Despite their effectiveness, existing consistency-based defenses still rely on loosely coupled, modular designs where visual and kinematic features are processed in separate pipelines and only fused at score or decision level [37,38]. This limits their ability to use physical consistency as a structural prior and leaves nontrivial safety gaps under adaptive physical attacks.

## 3. Proposed Algorithm

### 3.1. Method Overview

For clarity, the key symbols and abbreviations used throughout this paper are summarized in Table 1. To overcome the limitations of existing defenses in real-time performance, semantic preservation, and feature coupling, we propose a physics-aware trajectory–appearance consistency framework. Rather than relying on hand-crafted consistency checks or loosely coupled pipelines, the framework learns the nonlinear coupling between visual semantics and physical motion within a unified computation graph.

**Figure 2:**
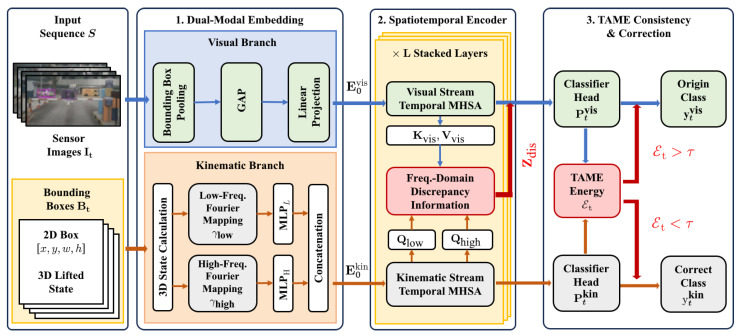
Overall architecture of the proposed physics-aware trajectory–appearance consistency defense framework.

Given a continuous observation sequence $S={\left\{\left(I_{t},B_{t}\right)\right\}}_{t=1}^{T}$, where $I_{t}$ is the raw image at time *t* and $B_{t}$ denotes 2D bounding boxes with lifted 3D coordinates, the framework proceeds in three stages, as shown in Figure 2. First, a dual-modal feature embedding module maps deep visual features and structured kinematic states into a shared latent space. The kinematic branch adopts a frequency-domain design that separately encodes low-frequency inertial trends and high-frequency jitter, yielding multi-scale motion embeddings for subsequent reasoning (Section 3.2). Second, a dual-stream spatiotemporal encoder, instantiated as a lightweight Transformer, jointly processes the visual and kinematic sequences. Within each layer, temporal self-attention aggregates long-range context in each stream, and frequency-domain cross-attention implements an *endogenous feature orchestration* mechanism: low-frequency Inertial Queries and high-frequency Jitter Queries retrieve two appearance patterns from the visual stream, whose discrepancy is distilled into a fused signal $Z_{dis}$ and injected back into the visual features via residual connections (Section 3.3). This layer-wise process produces consistency-aware contextual representations that encode how well appearance and trajectory agree over time. Third, a TAME head attaches classification heads to the final visual and kinematic representations to obtain $P_{t}^{vis}$ and $P_{t}^{kin}$, and defines the (TAME) energy as a differentiable measure of physical–visual conflict (Section 3.4).

At each time step *t*, the model outputs a tuple $\left({\hat{y}}_{t},E_{t}\right)$ for downstream safety decisions. Specifically, ${\hat{y}}_{t}$ is the object label selected by a TAME-based switching rule: when $E_{t}\leq \tau$, the appearance-based prediction is trusted; when $E_{t}>\tau$, the system overrides the visual decision with the motion-based prior. The scalar $E_{t}$ thus acts both as an attack confidence score and as a switch for semantic correction. The overall inference procedure is summarized in Algorithm 1.
**Algorithm 1** Inference of the dual-stream consistency defense (dual-frequency retrieval + TAME correction)**Require:** 
Image sequence ${\left\{I_{t}\right\}}_{t=1}^{T}$;  1:Detector D;  2:Dual-stream spatiotemporal encoder ${\mathrm{Enc}}_{\theta }$ (*L* layers);  3:Fourier matrices $M_{\mathrm{low}},M_{\mathrm{high}}$;  4:MLPs ${\mathrm{MLP}}_{L},{\mathrm{MLP}}_{H}$;  5:TAME threshold τ.**Ensure:** 
Corrected labels $\hat{Y}={\left\{{\hat{y}}_{t}\right\}}_{t=1}^{T}$; energies $E={\left\{E_{t}\right\}}_{t=1}^{T}$.  6:**Stage 1: Dual-Modal Embedding**  7:**for** t=1 to *T* **do**  8:   $\left(F_{map,t},B_{t}\right)\leftarrow D\left(I_{t}\right)$  9:   $e_{t}^{vis}\leftarrow \mathrm{VisualEmbed}\left(F_{map,t},B_{t}\right)\in R^{d}$10:   $s_{t}\leftarrow \mathrm{KinematicState}\left(B_{1:t}\right)\in R^{9}$   ▹$\left[x,y,l,w,h,v_{x},v_{y},a_{x},a_{y}\right]$11:   $\gamma_{\mathrm{low}}\left(s_{t}\right)\leftarrow \left[\cos\left(2\pi M_{\mathrm{low}}s_{t}\right),\sin\left(2\pi M_{\mathrm{low}}s_{t}\right)\right]$12:   $\gamma_{\mathrm{high}}\left(s_{t}\right)\leftarrow \left[\cos\left(2\pi M_{\mathrm{high}}s_{t}\right),\sin\left(2\pi M_{\mathrm{high}}s_{t}\right)\right]$13:   $e_{t}^{kin}\leftarrow \mathrm{Concat}\;\left({\mathrm{MLP}}_{L}\left(\gamma_{\mathrm{low}}\right),\;{\mathrm{MLP}}_{H}\left(\gamma_{\mathrm{high}}\right)\right)\in R^{d}$14:**end for**15:$E_{0}^{vis}\leftarrow {\left[e_{t}^{vis}\right]}_{t=1}^{T}\in R^{T\times d}$, $E_{0}^{kin}\leftarrow {\left[e_{t}^{kin}\right]}_{t=1}^{T}\in R^{T\times d}$16:**Stage 2: Dual-Stream Spatiotemporal Encoding with Endogenous Orchestration**17:$\left(E_{L}^{vis},E_{L}^{kin}\right)\leftarrow {\mathrm{Enc}}_{\theta }\left(E_{0}^{vis},E_{0}^{kin}\right)$  ▹ per layer: MHSA + dual-frequency cross-attn $\to Z_{low},Z_{high}$; $\Delta Z=Z_{low}-Z_{high}$; inject $Z_{dis}$18:**Stage 3: TAME Check and Physics-Guided Correction**19:**for** t=1 to *T* **do**20:   $P_{t}^{vis}\leftarrow \mathrm{Softmax}\left(W_{c}^{vis}\;E_{L}^{vis}\left[t\right]+b_{c}^{vis}\right)$21:   $P_{t}^{kin}\leftarrow \mathrm{Softmax}\left(W_{c}^{kin}\;E_{L}^{kin}\left[t\right]+b_{c}^{kin}\right)$22:   $E_{t}\leftarrow D_{\mathrm{KL}}\left(P_{t}^{kin}\|P_{t}^{vis}\right)+D_{\mathrm{KL}}\left(P_{t}^{vis}\|P_{t}^{kin}\right)$23:   ${\hat{y}}_{t}\leftarrow \left\{\begin{array}{cc} \arg\max_{y}P_{t}^{kin}\left(y\right), & E_{t}>\tau \\ \arg\max_{y}P_{t}^{vis}\left(y\right), & \mathrm{otherwise} \end{array}\right.$24:**end for**25:**return** $\hat{Y},E$

### 3.2. Dual-Modal Feature Embedding

This module maps unstructured visual information and structured kinematic data into a unified latent space $R^{d}$, enabling end-to-end interaction between heterogeneous modalities.

**Visual semantic embedding.** We reuse the backbone of the object detector to extract deep semantic features, avoiding redundant computation while retaining high-level information [23]. Given an input image $I_{t}$ and the corresponding bounding boxes $B_{t}$ at time *t*, we first obtain an intermediate feature map $F_{map}$. Region-of-interest features are then extracted by pooling over the bounding box locations and compressed into a feature vector using Global Average Pooling (GAP). A learnable linear projection $W_{vis}$ maps the pooled feature to the target dimension *d*:

(1) $$e_{t}^{vis}=W_{vis}\cdot \mathrm{GAP}\left(\mathrm{Pooling}\left(F_{map},B_{t}\right)\right).$$

The embedding $e_{t}^{vis}$ encodes texture, shape, and category-level semantics, and later serves as Keys and Values in the cross-attention with kinematic queries [39].

**Frequency-domain kinematic embedding.** In real-world driving, low-frequency trajectories (e.g., smooth velocity profiles) capture the coarse motion of objects, while high-frequency fluctuations often reflect sensor noise and tracking instability [20,40]. To capture both aspects, we introduce a frequency-domain motion embedding inspired by Fourier feature mappings [41]. This design separately encodes low-frequency inertial trends and high-frequency jitter, providing richer evidence for trajectory–appearance consistency.

Each instance is associated with the 3D bounding box of the object. We compute the centroid $p_{t}$ as the mean of the eight corners and use its $\left(x_{t},y_{t}\right)$ coordinates on the ground plane, ignoring the vertical coordinate due to its limited variation and high noise [17]. Given a frame rate $f_{r}$, the instantaneous velocity $v_{\alpha ,t}$ and acceleration $a_{\alpha ,t}$ along axis α are obtained via finite differences:

(2) $$v_{\alpha ,t}=f_{r}\left[p_{\alpha ,t}-p_{\alpha ,t-1}\right],\;\alpha \in \left\{x,y\right\},$$

(3) $$a_{\alpha ,t}=f_{r}^{2}\left[p_{\alpha ,t}-2p_{\alpha ,t-1}+p_{\alpha ,t-2}\right],\;\alpha \in \left\{x,y\right\}.$$

We then construct a compact physical state vector $s_{t}\in R^{9}$ as [17]:

(4) $$s_{t}={\left[x_{t},y_{t},l_{t},w_{t},h_{t},v_{x,t},v_{y,t},a_{x,t},a_{y,t}\right]}^{⊤}.$$

To parameterize motion at different temporal frequencies, we apply learnable Fourier feature mappings:

(5) $$\gamma_{low}\left(s_{t}\right)={\left[\cos\left(2\pi M_{low}s_{t}\right),\;\sin\left(2\pi M_{low}s_{t}\right)\right]}^{⊤},$$

(6) $$\gamma_{high}\left(s_{t}\right)={\left[\cos\left(2\pi M_{high}s_{t}\right),\;\sin\left(2\pi M_{high}s_{t}\right)\right]}^{⊤},$$

where $M_{low}∼N\left(0,\sigma_{low}^{2}\right)$ encodes smooth inertial trends and $M_{high}∼N\left(0,\sigma_{high}^{2}\right)$ with $\sigma_{high}\gg \sigma_{low}$ captures higher-frequency jitter.

The two frequency components are processed by separate Multi-Layer Perceptrons (MLPs) and concatenated to form the final kinematic embedding:

(7) $$e_{t}^{kin}=\mathrm{Concat}({\mathrm{MLP}}_{L}\left(\gamma_{low}\left(s_{t}\right)\right),\;{\mathrm{MLP}}_{H}\left(\gamma_{high}\left(s_{t}\right)\right)).$$

By decoupling low- and high-frequency components, the encoder retains crucial jitter signals, enhancing the sensitivity of the downstream endogenous feature orchestration and TAME metric to adversarial perturbations, especially under adaptive attacks that primarily manipulate low-frequency trajectories.

### 3.3. Dual-Stream Spatiotemporal Encoder with Endogenous Feature Orchestration

**Figure 3:**
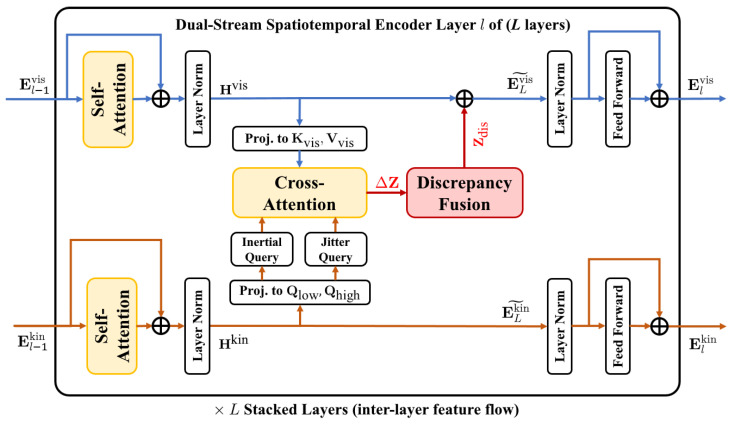
Structure of the dual-stream spatiotemporal encoder with endogenous feature orchestration.

The core reasoning unit of our framework is a dual-stream spatiotemporal encoder that captures temporal continuity within each modality and logical consistency across modalities. The encoder consists of *L* identical layers. At layer *l*, the inputs are the visual feature sequence $E_{l-1}^{vis}$ and the frequency-domain kinematic feature sequence $E_{l-1}^{kin}$. Each layer comprises two components: temporal self-attention in each stream and frequency-guided cross-attention with endogenous feature orchestration. The overall structure is illustrated in Figure 3.

**Temporal continuity modeling via self-attention.** In the physical world, both visual appearance and motion evolve smoothly over time. To model this continuity, we apply Multi-Head Self-Attention (MHSA) independently to the visual and kinematic streams. At each layer *l*, $E_{l-1}^{vis}$ and $E_{l-1}^{kin}$ are processed to obtain temporally contextualized features $H^{vis}$ and $H^{kin}$, which aggregate long-range context in each modality and provide stable inputs for cross-modal reasoning.

**Endogenous feature orchestration via frequency-domain retrieval.** Beyond separate temporal modeling, we use kinematics as an *internal organizer* of visual representations. Leveraging the frequency-domain embeddings from Section 3.2, each layer constructs a low-frequency Inertial Query $Q_{low}$ and a high-frequency Jitter Query $Q_{high}$ from the kinematic stream, and uses them to attend to the visual stream. These queries retrieve two appearance patterns, $Z_{low}$ and $Z_{high}$, that explain the observed motion under different spectral viewpoints.

The disparity between these two retrieved patterns,

(8) $$\Delta Z=Z_{low}-Z_{high},$$

captures how consistently visual semantics are supported across inertial and jitter-aware motion cues. For benign objects, both queries typically converge to compatible semantic explanations, yielding small ΔZ. Under physical attacks, high-frequency jitter and mismatched dynamics induce conflicting retrievals, resulting in a large semantic gap.

To turn this gap into an internal control signal, we feed the concatenated triplet $\left(Z_{low},Z_{high},\Delta Z\right)$ into a lightweight Feed-Forward Network (FFN), denoted as ${\mathrm{FFN}}_{disc}$, and obtain a fused discrepancy code:

(9) $$Z_{dis}={\mathrm{FFN}}_{disc}\left(\mathrm{Concat}\left(Z_{low},Z_{high},\Delta Z\right)\right).$$

Rather than using $Z_{dis}$ as a separate detector, we treat it as an *endogenous feature orchestration* signal that reorganizes the visual stream. Concretely, $Z_{dis}$ is injected back into the visual features through residual connections and Layer Normalization, amplifying channels that are consistent with kinematic evidence and suppressing channels dominated by adversarial artifacts or sensor noise. The kinematic stream is updated independently to preserve physically grounded dynamics.

Through this recurrent interaction, each layer performs frequency-aware feature orchestration: kinematic queries probe the visual stream, measure trajectory–appearance discrepancy, and use the resulting discrepancy code to reshape the internal representation manifold. As shown in the ablation study, removing this discrepancy feedback significantly increases false positives, confirming that endogenous feature orchestration is crucial for stabilizing benign predictions and exposing adversarial inconsistencies.

### 3.4. Trajectory–Appearance Mutual Exclusion Energy

Let $E_{L}^{vis},E_{L}^{kin}\in R^{T\times d}$ denote the final-layer outputs of the encoder for the visual and kinematic streams, respectively. The row vectors $e_{L,t}^{vis}$ and $e_{L,t}^{kin}$ serve as time-wise contextual representations for constructing the TAME energy.

Built on the encoder’s layer-wise reasoning, $E_{L}^{vis}$ and $E_{L}^{kin}$ integrate three sources of information: (i) visual appearance cues, (ii) frequency-domain kinematic patterns, and (iii) discrepancy-sensitive corrections injected by the endogenous feature orchestration. The final representations thus encode the compatibility between visual appearance and physical dynamics, allowing us to define the TAME energy in this inconsistency-aware space.

To obtain semantic predictions from appearance and motion, we attach classification heads to the final representations. At each time step *t*, we compute class posterior distributions via softmax:

(10) $$P_{t}^{\alpha }\left(y\right)=\mathrm{Softmax}\;{\left(W_{c}^{\alpha }\;e_{L,t}^{\alpha }+b_{c}^{\alpha }\right)}_{y},\;\alpha \in \left\{\mathrm{vis},\mathrm{kin}\right\}.$$

Here, $W_{c}^{\alpha }$ and $b_{c}^{\alpha }$ denote the learnable weights and bias of the classifier. $P_{t}^{vis}\left(y\right)$ reflects the class distribution inferred from visual appearance, and $P_{t}^{kin}\left(y\right)$ reflects the motion-based prior.

Assuming that physical trajectories are harder to forge than appearance, we expect $P_{t}^{vis}$ and $P_{t}^{kin}$ to agree on benign samples and to diverge under physical attacks. We define the TAME energy at time *t* as the sum of the forward and reverse Kullback–Leibler divergences:

(11) $$E_{t}=D_{\mathrm{KL}}\;\left(P_{t}^{kin}\;∥\;P_{t}^{vis}\right)+D_{\mathrm{KL}}\;\left(P_{t}^{vis}\;∥\;P_{t}^{kin}\right),$$

with a small constant ϵ (e.g., $10^{-8}$) added for numerical stability:

(12) $$D_{\mathrm{KL}}\;\left(P_{t}^{a}\;∥\;P_{t}^{b}\right)=\sum_{y\in Y}P_{t}^{a}\left(y\right)\;\log\frac{P_{t}^{a}\left(y\right)}{P_{t}^{b}\left(y\right)+\epsilon }.$$

When appearance and motion are compatible (e.g., a vehicle-like appearance and a high-speed trajectory), both distributions concentrate on the same classes, leading to low TAME energy. In contrast, adversarial attacks cause a semantic conflict, and $E_{t}$ becomes large.

We use TAME for both attack detection and semantic correction. Given a threshold τ, the final prediction at time *t* is

(13) $${\hat{y}}_{t}=\left\{\begin{array}{cc} \arg\max_{y\in Y}P_{t}^{vis}\left(y\right), & E_{t}\leq \tau ,\\ \arg\max_{y\in Y}P_{t}^{kin}\left(y\right), & E_{t}>\tau . \end{array}\right.$$

Thus, ${\hat{y}}_{t}$ is an error-correcting prediction: it trusts the appearance-based decision when trajectory and appearance align and switches to the motion-based prior when a physical–visual inconsistency is detected.

### 3.5. Model Training and Inference

**Supervised classification.** Given a training sequence with ground-truth labels ${\left\{y_{t}\right\}}_{t=1}^{T}$, we compute $P_{t}^{vis}$, $P_{t}^{kin}$, and $E_{t}$ as in Equations (10) and (11). The primary supervision is a cross-entropy loss applied to both heads:

(14) $$L_{\mathrm{cls}}=\frac{1}{T}\sum_{t=1}^{T}(\mathrm{CE}\left(P_{t}^{vis},y_{t}\right)+\mathrm{CE}\left(P_{t}^{kin},y_{t}\right)),$$

which encourages both modalities to predict the correct class.

**Consistency regularization and adversarial calibration.** To shape the TAME energy landscape, we penalize large energy on clean samples and enforce a margin on adversarial ones. For clean data, a consistency term

(15) $$L_{\mathrm{con}}=\frac{1}{T}\sum_{t=1}^{T}E_{t}$$

pushes $P_{t}^{vis}$ and $P_{t}^{kin}$ to agree, forming a low-energy manifold for benign samples. When adversarial examples $\left(I_{t}^{adv},B_{t}^{adv}\right)$ are available, we further apply a hinge-style margin loss:

(16) $$L_{\mathrm{adv}}=\frac{1}{T}\sum_{t=1}^{T}\left(\mathrm{CE}\left(P_{t}^{kin},y_{t}\right)+\max\left(0,m-E_{t}\right)\right),$$

where m>0 is a margin hyperparameter. This term keeps the kinematic head aligned with the true class while pushing adversarial samples to high-energy regions.

**Overall objective and inference.** The full training loss combines the above components on clean and adversarial data:

(17) $$L=L_{\mathrm{cls}}^{\left(\mathrm{clean}\right)}+\lambda_{\mathrm{con}}\;L_{\mathrm{con}}^{\left(\mathrm{clean}\right)}+\lambda_{\mathrm{adv}}\;L_{\mathrm{adv}}^{\left(\mathrm{adv}\right)},$$

where $\lambda_{\mathrm{con}}$ and $\lambda_{\mathrm{adv}}$ control the strength of consistency regularization and adversarial calibration.

At inference, given $S={\left\{\left(I_{t},B_{t}\right)\right\}}_{t=1}^{T}$, we reuse the frozen detector backbone to obtain $F_{map}$, perform dual-modal embedding as in Section 3.2, and feed the resulting sequences into the dual-stream spatiotemporal encoder to obtain $E_{L}^{vis}$ and $E_{L}^{kin}$. The TAME head yields $P_{t}^{vis}$, $P_{t}^{kin}$, and $E_{t}$, and the decision rule in Equation (13) is applied with a threshold τ selected based on validation data. Since the detector backbone is reused and all additional modules are lightweight, the overall overhead is small, making the module suitable for real-time deployment behind existing object detectors.

## 4. Experiments

### 4.1. Experimental Setup

**Datasets.** We evaluate the proposed defense on three widely used autonomous driving benchmarks: KITTI [42], nuScenes [43], and BDD100K [44], which cover diverse driving environments and multimodal sensor data. These datasets cover a spectrum of driving complexities, ranging from the structured urban scenarios in KITTI and the multimodal sensor data in nuScenes to the large-scale, heterogeneous traffic environments in BDD100K. We focus on five representative traffic participants: *bicycle*, *bus*, *pedestrian*, *car*, and *truck*. nuScenes and KITTI provide 21,763 and 5212 object-specific clips, respectively. For BDD100K, we use its Multi-Object Tracking subset (1600 videos) and select approximately 5000 instances from our target categories. These datasets allow for a rigorous evaluation of defense performance under real-world conditions.

**Implementation Details.** For visual perception, we use a YOLOv8 [23] detector fine-tuned on BDD100K and keep its backbone frozen. During training, we run YOLOv8 and Simple Online and Realtime Tracking (SORT) [45] on BDD100K videos to obtain backbone feature maps, detection boxes/scores, and per-object trajectories ${\left(I_{t},B_{t}\right)}_{t=1}^{T}$, and optimize the consistency encoder and TAME head end-to-end on top of these signals using AdamW with an initial learning rate of $3\times 10^{-4}$, weight decay of $1\times 10^{-4}$, and batch size of 32, which were optimized to ensure stable convergence on the validation set. We train for 60 epochs with a cosine learning-rate schedule and a warm-up of 5 epochs. The encoder has L=3 layers, hidden dimension d=256, and 8 attention heads per layer. Hyperparameters were determined through sensitivity analysis on the validation set to balance defense effectiveness and training stability: the loss weights were set to $\lambda_{\mathrm{con}}=0.1$ and $\lambda_{\mathrm{adv}}=1.0$, assigning a lower weight to $\lambda_{\mathrm{con}}$ to prevent regularization from dominating early training while ensuring sufficient penalty on adversarial samples via $\lambda_{\mathrm{adv}}$. The TAME margin was set to m=0.9 to enforce a significant energy gap between benign and adversarial manifolds. Finally, the decision threshold τ=0.2 was determined via quantitative trade-off analysis, aiming to maximize Detection Accuracy (DA) while strictly bounding the false positive rate (FPR) below 5% in benign scenarios. At inference time, this trained module is reused as a plug-in safety layer without retraining. All experiments run on 2 NVIDIA RTX A6000 GPUs (NVIDIA Corporation, Santa Clara, CA, USA) with 48 GB memory.

### 4.2. Attack Configuration

We focus on physically realizable adversarial attacks, as modifying the surfaces of traffic participants is a tangible threat. The perturbation mask is constrained within the target’s physical boundaries to ensure realism. We evaluate three patch scales, large (100×100), medium (80×80), and small (60×60), optimized under $L_{p}$-norm and NPS constraints.

We use three representative attack methods:

- **RP2** [8]: Generates robust physical perturbations to induce misclassification under varying conditions.
- **CAPatch** [34]: Adapted from image captioning, it maximizes detection errors in autonomous driving contexts.
- **SLAP** [35]: A projector-based optical attack simulating light-based perturbations.

**Figure 4:**
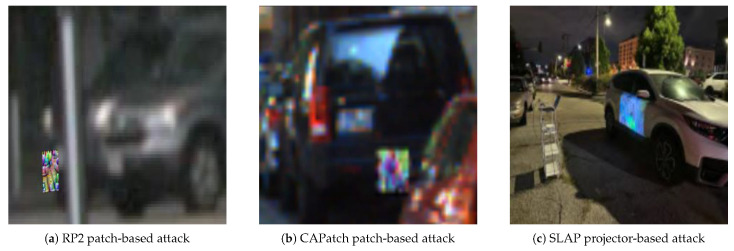
Illustration of physical adversarial attacks on autonomous driving perception: (**a**) RP2 patch-based attack on traffic signs or vehicles; (**b**) CAPatch-style patch attack adapted to detection scenarios; (**c**) SLAP optical projection attack on object surfaces.

These attacks are applied to the selected object categories. We simulate dynamic attacks using ground-truth 3D poses and adjust the patch’s homography frame-by-frame, ensuring realistic appearance changes during motion. To intuitively understand these threats, visual examples of the RP2, CAPatch, and SLAP attacks applied to our target datasets are illustrated in Figure 4.

### 4.3. Evaluation Metrics

To evaluate defense effectiveness, we use metrics that assess detection ability, correction ability, false alarms, and efficiency.

**Detection Accuracy (DA).** DA reflects the ability of a defense to identify misclassified instances caused by attacks:

(18) $$\mathrm{DA}=\frac{N_{\det}}{N_{\mathrm{mis}}}$$

**Correction Accuracy (CA).** CA measures the ability of a defense to recover the correct label once an attack has occurred:

(19) $$\mathrm{CA}=\frac{N_{\mathrm{corr}}}{N_{\mathrm{mis}}}$$

**False Positive Rate (FPR).** FPR characterizes the risk that benign samples are incorrectly treated as attacked by the defense:

(20) $$\mathrm{FPR}=\frac{N_{\mathrm{fp}}}{N_{\mathrm{benign}}}$$

**False Negative Rate (FNR).** FNR measures the proportion of truly attacked samples that are still misclassified after applying the defense, i.e., the missed attacks of the defense:

(21) $$\mathrm{FNR}=\frac{N_{\mathrm{fn}}}{N_{\mathrm{mis}}}$$

**Runtime Efficiency (RE).** RE evaluates whether a defense satisfies real-time constraints. Let $t_{i}$ denote the end-to-end processing time of the *i*-th sample and *n* the total number of samples. The average runtime per sample is:

(22) $$\mathrm{RE}=\frac{1}{n}\sum_{i=1}^{n}t_{i}\;\;$$

### 4.4. Baselines

To validate the effectiveness, we compare it with five representative defenses covering input purification, certified robustness, and spatiotemporal consistency modeling. These baselines include both state-of-the-art general defense strategies and physics-aware approaches in autonomous driving.

**DiffPure** [13] is an input purification method that uses pre-trained diffusion models to sanitize adversarial examples. While effective in removing perturbations, it may degrade high-frequency semantic details necessary for small object recognition.

**PatchGuard** [11] provides certified robustness against localized adversarial patches. It uses small receptive fields and robust aggregation mechanisms to limit feature corruption, but its high computational overhead restricts real-time object detection.

**DetectorGuard** [46] secures object detectors against patch-hiding attacks. It cross-references the detector’s output with a robust objectness predictor to detect inconsistencies. However, it focuses more on object presence than spatiotemporal dynamics.

**PercepGuard** [16] uses spatiotemporal consistency to detect misclassification attacks. It employs a Recurrent Neural Network (RNN) to classify 2D bounding boxes and flags alarms when the trajectory-inferred class contradicts the visual detection. However, it filters out high-frequency jitter, limiting robustness against adaptive attacks.

**PhySense** [17] is a physics-aware defense that integrates features like texture, dynamic behavior, and inter-object interactions. While comprehensive, its loose coupling of feature extraction modules leads to significant latency and fails to fully capture correlations between visual and kinematic modalities.

### 4.5. Defense Performance

We first evaluate the defense performance of the proposed defense against RP2, CAPatch, and SLAP on nuScenes, KITTI, and BDD100K, each with three patch scales (large, medium, small). As shown in Table 2, the proposed defense consistently outperforms PhySense across almost all attack types, patch sizes, and datasets. In most configurations, our DA is comparable to or slightly higher than that of PhySense, while CA improves by a clear margin and FPR/FNR are typically reduced across datasets and patch sizes. In a few relatively easy KITTI settings, PhySense attains marginally higher DA, but ours still achieves much higher CA and significantly lower FPR/FNR, indicating a strictly better robustness–utility trade-off.

**Effect of patch size and attack type.** As the patch size shrinks from large to small, both ours and PhySense exhibit the expected degradation in DA and CA due to the increased visual stealthiness and reduced footprint of the adversarial patch. CA is consistently higher and FPR/FNR are generally lower than PhySense across datasets and patch sizes, with only minor deviations in a few easy settings. This trend is especially salient under SLAP, the projector-based optical attack that induces rapid, transient appearance changes. On nuScenes with small SLAP patches, for instance, our method raises CA from 0.728 to 0.835 and cuts FNR by more than half, showing that the TAME energy is sensitive to physically inconsistent motion even when visual perturbations are small and short-lived.

**Comparison with baselines.** Table 3 further positions our method against a broader spectrum of defenses on nuScenes under RP2 with large patches. Input purification (DiffPure) and certified patch defenses (PatchGuard) provide useful robustness guarantees but either incur high false alarms on benign samples or struggle to maintain correction performance in realistic detection settings. Detector-oriented defenses (DetectorGuard) and trajectory-only methods (PercepGuard) capture parts of the physical picture but still leave a considerable gap in either DA, CA, or FPR. PhySense, as a strong physics-aware baseline, narrows this gap by integrating multiple hand-crafted physical cues, yet it still operates under a loosely coupled, modular architecture. In contrast, our method achieves leading performance across all metrics, supporting the benefits of deeply coupled, frequency-guided trajectory–appearance reasoning.

**Runtime analysis.** In terms of RE, we reuse the frozen detector backbone and rely only on Transformer-style operations without external hand-crafted feature extractors. As shown in Table 2, the per-frame overhead of PhySense ranges from about 0.028 s to 0.043 s across datasets, whereas our method remains in the 0.015–0.019 s range. Thus, our method achieves stronger robustness and better calibration of physical inconsistency while still meeting real-time constraints in autonomous driving deployments.

### 4.6. Black-Box Transferability

We further examine how well the proposed defense transfers in a realistic setting, where the safety module is trained once and then reused across heterogeneous detectors, attacks, and datasets. Using the defense module trained as described in Section 4.1, we then evaluate this single model under three settings: (i) changing the base detector to Faster R-CNN [47] or CenterNet [48], (ii) changing the dataset to nuScenes or KITTI, and (iii) changing the attack family to CAPatch or SLAP, still with medium patches. Table 4 summarizes the results. The configuration corresponds to the training setting, while all other entries represent zero-shot transfer without any re-training of the defense module.

**Cross-detector transfer.** On BDD100K under RP2, replacing YOLOv8 with Faster R-CNN or CenterNet leads to only a small drop in DA and CA, and a slight increase in FPR/FNR. The overall performance remains in a similar range as the original YOLOv8-based configuration. This indicates that the dual-stream spatiotemporal encoder and TAME head indeed behave as a detector-agnostic safety layer: as long as bounding boxes, labels, and trajectories are available, the module can be plugged behind different detectors without re-training, while still providing substantial gains over PhySense and other baselines (Table 2).

**Cross-attack and cross-dataset transfer.** Using the same model and threshold, we then change both the dataset and the attack type. Across nuScenes and KITTI, and for RP2, CAPatch, and SLAP, YOLOv8-based results show only modest degradation in DA/CA compared with the in-domain BDD100K–RP2 configuration, while FPR/FNR remain low. The trends are similar when switching to Faster R-CNN or CenterNet: although absolute performance slightly decreases due to detector- and domain-specific differences, the defense remains effective across all combinations. Notably, the model retains strong correction ability against CAPatch and SLAP even though it was adversarially calibrated on RP2, suggesting that the frequency-domain kinematic embedding and TAME-based inconsistency reasoning capture generic trajectory–appearance discrepancies instead of overfitting to a single patch pattern or dataset.

Overall, the results in Table 4 show that a single trained module can be transferred across heterogeneous perception stacks and deployment scenarios, with only limited loss of robustness. This transferability is particularly attractive for large-scale autonomous driving systems, where maintaining one bespoke safety module per detector or per fleet would be impractical.

### 4.7. Defense Against Adaptive Attackers

We finally evaluate the proposed defense against adaptive attackers that are aware of the trajectory–appearance consistency checks and attempt to jointly fool both the detector and the defense.

#### 4.7.1. Attacker Knowledge and Goals

We consider a strong white-box threat model in which the attacker has access to the architecture and parameters of both the base detector and the module. (We assume no access to the validation set used to select the TAME threshold and no control over the tracking pipeline.) The adversary optimizes a physically realizable patch as in Section 4.2, under the same constraints on patch size, location, and NPS. The goal is two-fold: (i) induce a targeted misclassification by the detector and (ii) keep the TAME energy $E_{t}$ below the detection threshold τ, so that the defense neither raises an alarm nor corrects the label. In other words, the attacker seeks perturbations that jointly maximize detector loss on the target class and minimize $E_{t}$ or its contributing terms.

#### 4.7.2. Adaptive Attack Strategies

We instantiate this threat model with three representative strategies that exploit progressively more internal details:

- **Trajectory-Smoothing RP2.** The standard RP2 loss is augmented with a smoothness regularizer on the sequence of 2D/3D bounding boxes, penalizing frame-to-frame variations in velocity and acceleration. This encourages low-frequency, inertial-like trajectories but does not directly optimize TAME.
- **TAME-Aware Joint Optimization.** The attacker differentiates through the dual-stream encoder and TAME head. The patch is optimized to (a) drive the visual head $P_{t}^{\mathrm{vis}}$ toward a target class $y^{\mathrm{adv}}$ and (b) reduce the symmetric TAME energy so that $P_{t}^{\mathrm{vis}}$ and $P_{t}^{\mathrm{kin}}$ agree on $y^{\mathrm{adv}}$:
  (23) $$L_{\mathrm{adv}}^{\mathrm{TAME}}=L_{\det}\left(y^{\mathrm{adv}}\right)+\beta \;E_{t}\left(P_{t}^{\mathrm{vis}},P_{t}^{\mathrm{kin}}\right),$$
  where β balances misclassification and energy suppression.
- **Frequency-Suppression Attack.** Assuming knowledge of the frequency-decoupling mechanism, the attacker penalizes the magnitude of the high-frequency component $\gamma_{\mathrm{high}}\left(s_{t}\right)$:
  (24) $$L_{\mathrm{freq}}=∥\gamma_{\mathrm{high}}\left(s_{t}\right)-\gamma_{\mathrm{low}}\left(s_{t}\right){∥}_{2}^{2},$$
  aiming to suppress jitter-related responses in the kinematic stream while still fooling the detector.

#### 4.7.3. Results and Analysis

As summarized in Table 5, we present the defense performance on nuScenes under adaptive attackers.

The Trajectory-Smoothing strategy reduces CA from 0.921 to 0.786 by making 3D box sequences closer to the ideal inertial motion, but the drop is moderate, as the frequency-domain embedding still captures residual discrepancies. The TAME-aware attack is the most effective, lowering CA to 0.724 and increasing FNR to 0.169, showing that a fully informed attacker can sometimes force the two heads to agree on wrong labels. The Frequency-Suppression attack achieves similar CA (0.725): suppressing jitter weakens the high-frequency cue but inevitably distorts low-frequency motion, which remains detectable.

Overall, these results expose a fundamental dilemma for adaptive attackers. To reliably fool the base detector, the patch must introduce persistent appearance changes that create additional jitter and trajectory–appearance mismatch, pushing the TAME energy $E_{t}$ upward. To evade TAME, the attacker must instead smooth motion and suppress jitter, which weakens the perturbation and undermines the misclassification. Because our frequency-domain kinematic embedding defines robustness in terms of the contrast between inertia and jitter rather than any single trajectory statistic, lowering $E_{t}$ by manipulating one band typically worsens the other; so in practice, adaptive optimization can at best move sequences from the high-energy region to a narrow band around τ, rather than back to the benign low-energy manifold.

### 4.8. Scene-Level Behavior and Consistency Landscape

Beyond aggregate metrics, we analyze how the proposed trajectory–appearance consistency behaves at the scene and trajectory level. All visualizations in this subsection are produced on held-out nuScenes sequences; the observations are representative of the trends seen on other datasets.

**Figure 5:**
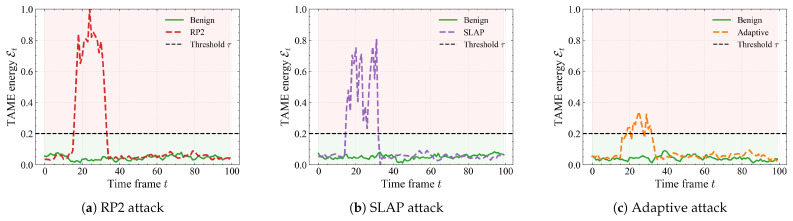
Trajectory-level evolution of the TAME energy $E_{t}$ under different physical attack types. Each panel compares a benign trajectory (green) with an attacked one (colored curve), with the horizontal dashed line indicating the detection threshold τ=0.2. The proposed defense responds to RP2 and SLAP by producing persistent high-energy segments, while adaptive attacks succeed in partially suppressing the peak but still incur noticeable deviations from the benign profile.

**Frame-wise energy evolution.** As illustrated in Figure 5, we plot the TAME energy $E_{t}$ over time for three typical sequences under RP2, SLAP, and adaptive attacks, together with the benign counterpart. For benign trajectories (green curves), $E_{t}$ stays close to a low baseline around 0.05 and rarely approaches the decision threshold τ=0.2, indicating that appearance and motion remain compatible over the whole sequence. Once an RP2 patch becomes effective (frames 15–35), the energy quickly rises into a high plateau (≈0.6–0.9) and remains above the shaded alarm region, clearly separating attacked frames from clean ones. SLAP produces a similar but more oscillatory plateau, reflecting the transient nature of projector-based perturbations. In the adaptive case, where the attacker explicitly tries to keep $E_{t}$ small, the curve oscillates tightly around τ instead of returning to the benign baseline, showing that it is difficult to simultaneously fool the detector and keep the trajectory on the low-energy manifold defined in Section 3.4.

**Figure 6:**
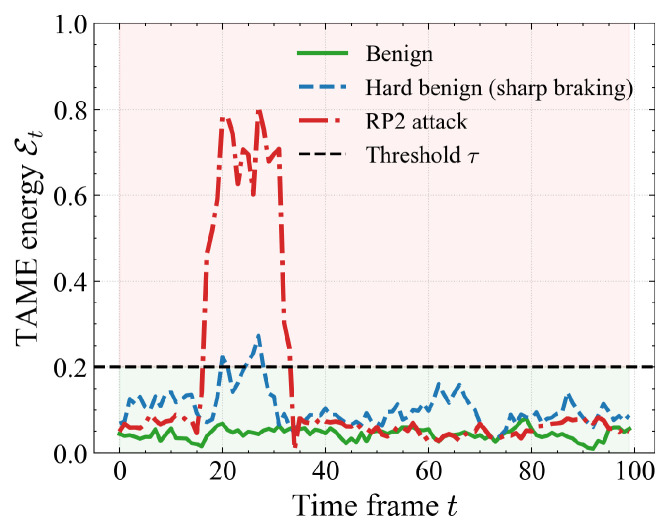
Comparison of TAME energy $E_{t}$ over time for a normal benign trajectory, a hard-benign scenario (sharp braking), and an RP2 attack. Although sharp braking transiently increases $E_{t}$ towards the threshold τ, its peak remains lower and much shorter than the sustained high-energy plateau induced by RP2, illustrating that our defense can distinguish physically plausible maneuvers from genuine adversarial instability.

To examine potential false alarms, as shown in Figure 6, we compare a benign trajectory, a “hard benign” case with sharp braking, and an RP2 attack. Sharp braking temporarily increases $E_{t}$ and produces a short bump that touches or slightly crosses the threshold, but quickly falls back to the benign band. In contrast, RP2 induces a long, high plateau that stays far above τ. This difference explains why the defense maintains a low FPR while still detecting physically inconsistent attacks.

**Figure 7:**
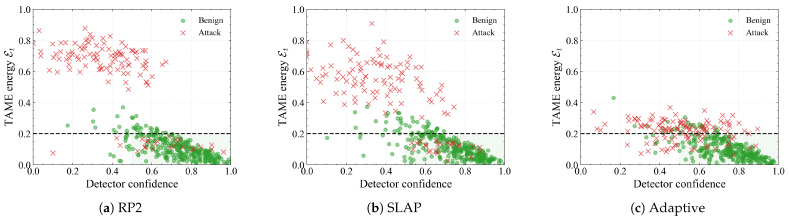
Joint distribution of detector confidence and TAME energy $E_{t}$ under different attack strategies. Green dots denote benign samples and red crosses indicate attacked samples. The shaded region in the lower-right corner represents the ideal operating regime (high confidence and low energy), while the horizontal dashed line marks the decision threshold τ. RP2 and SLAP mainly push samples into a high-energy band, whereas adaptive attacks concentrate around τ with moderately reduced confidence, confirming that our defense reshapes the score space into a physically calibrated separation between clean and adversarial states.

**Consistency vs. detector confidence.** As illustrated in Figure 7, we present scatter plots of TAME energy versus detector confidence for benign and attacked samples under RP2, SLAP and adaptive attacks. Benign detections (green dots) cluster in the lower-right region: high confidence and low energy, which corresponds to predictions that are both visually confident and physically plausible. RP2 and SLAP attacks (red crosses) mainly occupy the upper-right and upper-middle area: the base detector is still reasonably confident, but the TAME energy is well above τ, revealing strong trajectory–appearance conflict. Under adaptive attacks, adversarial samples move closer to the threshold and their confidence decreases slightly, yet they still form a distinct high-energy cloud separated from benign points. These plots confirm that $E_{t}$ provides information complementary to detector confidence: it exposes “high-confidence but physically inconsistent” cases that cannot be filtered by confidence alone.

**Figure 8:**
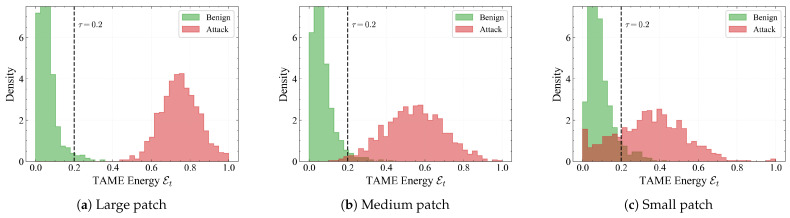
Distribution of TAME energy $E_{t}$ for benign and attacked trajectories under different patch sizes. Each panel plots kernel-smoothed histograms of benign (green) and adversarial (red) samples, with the vertical dashed line indicating the global threshold τ=0.2. Large patches lead to a clear bimodal separation, whereas smaller patches shift the attack distribution leftwards and increase overlap with benign tails, explaining the gradual increase in FNR observed in Table 2.

**Energy distributions across patch size and object class.** As shown in Figure 8, we report the marginal distributions of $E_{t}$ for benign and attacked frames under large, medium and small patches. For large patches, benign and attack distributions are almost disjoint: benign frames concentrate well below τ, whereas attacks form a broad peak around 0.7–0.8. As the patch shrinks, the attack distribution gradually shifts towards the threshold and slightly overlaps with the benign tail, reflecting the increased visual stealthiness of smaller perturbations. Even for small patches, however, the main attack mass remains on the high-energy side of τ, which is consistent with the low FNR observed in Table 2.

**Figure 9:**
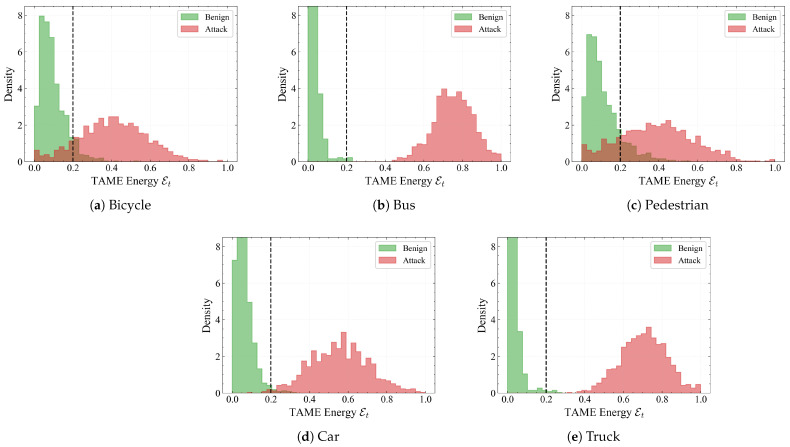
Class-wise TAME energy $E_{t}$ distributions for key traffic participants under RP2 attacks. Heavy vehicles such as buses and trucks exhibit very low benign energies and clearly separated high-energy attack modes, making them easier to defend. In contrast, bicycles and pedestrians show broader benign tails with substantial overlap around the threshold τ, reflecting intrinsically jittery motion patterns and explaining the slightly higher FNR on these categories. The black dashed vertical line marks the decision threshold τ=0.2, below which samples are considered benign and above which they are flagged as potential attacks.

Finally, as shown in Figure 9, we decompose the TAME distributions by object category (bicycle, bus, pedestrian, car, and truck). Across all classes, benign samples exhibit a sharp peak near zero and only a light tail around the threshold, indicating that the consistency prior is not biased towards a specific category. Attack distributions are shifted to higher energies, with large separation for buses and trucks (whose motion is more inertial) and slightly broader overlap for bicycles and pedestrians (which naturally move more erratically). Importantly, a single global threshold τ=0.2 still separates most benign and adversarial frames in every class, supporting the use of a class-agnostic decision rule in Equation (13) and explaining why the defense achieves stable performance across heterogeneous traffic participants.

### 4.9. Ablation Study

#### 4.9.1. Analysis of Deep-Coupling Mechanisms

We first examine the necessity of the high-order interactions modeled by the dual-stream spatiotemporal encoder. To this end, we contrast our fully coupled architecture with variants that represent typical designs. The quantitative comparison results are listed in Table 6.

The loose coupling variant follows the conventional pipeline in which visual and kinematic features are processed independently and only concatenated at the classification head. This failure confirms that correcting subtle inconsistencies requires early feature-level interaction to actively attenuate compromised visual cues. Replacing our frequency-domain design with a unified query (Single Q) noticeably degrades detection on jitter-heavy attacks such as SLAP, proving that a coarse motion representation fails to probe high-frequency adversarial artifacts. Furthermore, ablating the discrepancy feedback loop (w/o Discrepancy) spikes FPR, demonstrating that $Z_{dis}$ acts as a necessary stabilizer to suppress ambiguous features in benign scenes. Finally, the failure of frame-wise reasoning (w/o Self-Attn) under transient attacks underscores the necessity of temporal self-attention for capturing dynamic inconsistencies.

#### 4.9.2. Impact of Frequency-Domain Kinematic Embedding

We evaluate the spectral kinematic components in Table 7. The baseline (No Fourier) utilizing raw states underperforms, indicating that a single MLP fails to fully exploit spectrally localized cues. Crucially, discarding jitter information (w/o High Freq) results in high FNR, confirming that high-frequency fluctuations are strong discriminators for adversarial instability. Conversely, removing inertial context (w/o Low Freq) causes FPR to spike, showing that low-frequency trends are essential for stabilizing benign predictions against sensor noise. These observations are consistent with the hypothesis in Section 3.2 and justify the full frequency-domain design.

#### 4.9.3. Manifold Shaping via TAME Energy and Objectives

We analyze the decision manifold shaping in Table 8. (1) Benign Compression: Removing consistency regularization (w/o Con-Reg) causes a sharp rise in FPR, confirming that $L_{\mathrm{con}}$ is critical for compressing benign sequences into a compact low-energy manifold. (2) Adversarial Margin: Eliminating calibration (w/o Adv-Calib) significantly drops DA, proving that $L_{\mathrm{adv}}$ is necessary to explicitly push attacks into high-energy regions to ensure separability. (3) Metric Sensitivity: The inferiority of linear (L1 Distance) and asymmetric metrics (Asym. KL) highlights that Symmetric KL provides the necessary probabilistic sensitivity and steep gradients for decisive inconsistency detection.

## 5. Discussion

The experimental results validate our central hypothesis that physical adversarial attacks inevitably disturb the intrinsic coupling between visual appearance and motion, and that explicitly modeling this coupling in a shared latent space yields a more robust and efficient defense. Across three datasets, three attack families (RP2, CAPatch, SLAP), and multiple patch scales, our defense consistently improves DA and, more importantly, CA over PhySense while typically reducing FPR, FNR, and runtime. The scene-level visualizations further support this picture: benign trajectories remain on a compact low-energy manifold, whereas physical attacks induce sustained high-energy plateaus, and even adaptive attacks can only force the TAME curve to oscillate around the threshold instead of returning to the benign baseline (Figure 5, Figure 6, Figure 7, Figure 8 and Figure 9). Compared with certified defenses and input purification methods, our defense offers a different trade-off: rather than reconstructing clean images or providing formal but conservative guarantees, it leverages physically grounded consistency checks to achieve strong empirical robustness under strict real-time constraints.

The comparisons with spatiotemporal consistency-based methods further highlight the benefits of deep coupling. PercepGuard- and PhySense-style approaches already exploit trajectory information, yet they operate under loosely coupled, modular architectures and largely treat motion features as a post hoc auxiliary signal. In contrast, our method integrates visual and kinematic cues throughout the entire reasoning process via dual-modal self-attention and frequency-domain cross-attention. The confidence–energy scatter plots in Figure 7 reveal that attacked samples occupy a distinct high-energy band even when the detector is confident, providing a physically interpretable signal that complements conventional confidence scores. The transfer experiments in Table 4 further demonstrate that a single module trained on YOLOv8 with RP2 in BDD100K can be plugged behind Faster R–CNN and CenterNet and transferred to nuScenes and KITTI, as well as to unseen CAPatch and SLAP attacks, with only modest accuracy degradation and consistently low FPR/FNR. Together with the measured 15–19 ms per-frame overhead, this suggests that trajectory–appearance consistency can be deployed as a detector-agnostic safety layer in practical perception stacks.

The ablation studies provide additional insight into the mechanism of robustness. Removing temporal self-attention or reverting to loose coupling significantly degrades performance, confirming that inconsistency detection requires long-range temporal context and early interaction between modalities rather than simple score-level fusion. The frequency-domain kinematic embedding also proves crucial: dropping the high-frequency branch sharply increases FNR, whereas discarding low-frequency trends raises FPR, indicating that robustness emerges from the *relative* configuration of inertia and jitter rather than either component alone (Table 6 and Table 7). Patch-size and class-wise TAME histograms (Figure 8 and Figure 9) are consistent with the quantitative trends: smaller patches and intrinsically jittery participants such as bicycles and pedestrians exhibit larger overlap between benign and adversarial energies and correspondingly higher FNR, while heavy vehicles are much easier to separate.

Although implemented on RGB streams, the proposed Physics-Aware Spatiotemporal Consistency principle is fundamentally applicable to multimodal AV stacks. Since production perception systems often prioritize the visual branch for semantic classification in hybrid-fusion architectures [49], compromised visual inputs can propagate erroneous semantics to the fusion engine or trigger conservative failsafes. By sanitizing the visual branch at the feature level, our method effectively blocks this error propagation source.

Moreover, the framework provides resilience against second-order attack strategies. While sophisticated adversaries might attempt to jointly optimize appearance and trajectory to evade detection, our adaptive analysis (Section 4.7) exposes a fundamental stealthiness-dynamics dilemma: enforcing effective semantic misclassification inevitably induces high-frequency jitter or inertial violations [40]. Bypassing this defense in a multimodal setting would require satisfying kinematic constraints across all sensors simultaneously (e.g., aligning fake visual and LiDAR trajectories), imposing prohibitive optimization costs that render such attacks computationally infeasible or physically conspicuous. Future work will extend TAME to explicitly model cross-modal consistency (e.g., RGB-LiDAR flow alignment) to further heighten the barrier for adaptive threats.

Despite these advantages, our work is not a complete solution to physical adversarial threats. The framework assumes reasonably reliable tracking and 3D box lifting; severe tracking failures or sensor outages could impair the quality of kinematic features and thus the effectiveness of TAME. Moreover, our experiments focus on RGB-based perception and representative patch and projector attacks; other sensing modalities (e.g., LiDAR, radar), more complex multi-object attacks, and jointly optimized sensor-fusion strategies remain to be explored. Finally, our defense is trained and deployed with access to detector backbone feature maps. This relaxes a strict output-only black-box assumption, but allows us to reuse already computed features instead of running a separate visual backbone, substantially reducing computational overhead while preserving a detector-agnostic, plug-in interface. We view this as a deliberate trade-off between strict black-box constraints and the practical need to balance robustness, universality, and real-time efficiency in large-scale autonomous driving systems. These limitations point to important directions for future research on physically grounded, spatiotemporal defenses.

## 6. Conclusions

We presented a physics-aware trajectory–appearance consistency defense that treats physical trajectories not as an external verifier, but as an internal organizer of visual representations. By combining a dual-stream spatiotemporal encoder with endogenous feature orchestration and a frequency-domain kinematic embedding, the defense uses inertial trends and detection jitter to probe and modulate visual features, and it quantifies trajectory–appearance conflict via TAME energy. The resulting module can be attached as a transferable safety layer behind diverse object detectors by reusing their backbone features, outputs, and tracking states without modifying detector weights.

Extensive experiments on nuScenes, KITTI, and BDD100K show that the proposed defense substantially improves robustness against patch-based and projection-based physical attacks, achieving higher Correction Accuracy and typically lower FPR/FNR than prior consistency-based defenses such as PhySense, while reducing inference latency. The defense further exhibits strong cross-detector and cross-dataset transferability and maintains nontrivial protection under adaptive attackers. In future work, we plan to extend this trajectory–appearance consistency perspective to multi-sensor 3D perception, tighter integration with detection and tracking in closed-loop systems, and stronger adaptive benchmarks that jointly optimize over appearance and motion to further stress-test physically grounded defenses.

## Figures and Tables

**Table 1:** Nomenclature and abbreviations used in the proposed framework.

Symbol	Description
*Input and Embedding*	
*S*	Observation sequence ${\left\{\left(I_{t},B_{t}\right)\right\}}_{t=1}^{T}$
$I_{t},B_{t}$	Image and bounding boxes (2D box with lifted 3D state) at *t*
*T*	Temporal window length
$F_{map}$	Backbone feature map
$e_{t}^{vis}$	Visual semantic embedding ($\in R^{d}$)
*d*	Latent space dimension
$p_{t}$	Object centroid $\left(x_{t},y_{t}\right)$ on ground plane
$f_{r}$	Input frame rate
$s_{t}$	Kinematic state (position, velocity, acceleration)
$M_{low/high}$	Random Fourier projection matrices (low/high freq.)
$\gamma_{low/high}$	Fourier feature mappings (inertial/jitter)
$e_{t}^{kin}$	Kinematic embedding
*Dual-Stream Spatiotemporal Encoder*	
*L*	Number of encoder layers
$E_{l}^{vis/kin}$	Visual/Kinematic feature sequences at layer *l*
MHSA	Multi-Head Self-Attention
$Q_{low/high}$	Inertial (low-freq.) and Jitter (high-freq.) queries
$Z_{low/high}$	Retrieved appearance patterns via kinematic queries
ΔZ	Semantic discrepancy vector ($Z_{low}-Z_{high}$)
$Z_{dis}$	Fused discrepancy code for orchestration
*TAME Energy and Inference*	
$P_{t}^{vis/kin}$	Predicted class probabilities (visual/kinematic)
$D_{\mathrm{KL}}$	Kullback–Leibler divergence
$E_{t}$	Trajectory–Appearance Mutual Exclusion (TAME) energy
τ	Decision threshold
${\hat{y}}_{t}$	Final predicted label
*Optimization Objectives*	
$L_{\mathrm{cls}}$	Cross-entropy classification loss
$L_{\mathrm{con}}$	Consistency regularization loss
$L_{\mathrm{adv}}$	Adversarial calibration loss
*m*	Margin for adversarial calibration
$\lambda_{\mathrm{con}/\mathrm{adv}}$	Loss weighting coefficients

**Table 2:** Defense performance against RP2, CAPatch, and SLAP attacks. **Bolded** values in the table indicate optimal performance results.

Attack	Patch	Dataset	Frames	PhySense (Baseline)						Ours				
				DA	CA	FPR	FNR	RE		DA	CA	FPR	FNR	RE
RP2	Large	nuScenes	21,763	0.915	0.865	0.088	0.071	0.042		**0.935**	**0.921**	**0.041**	**0.035**	**0.019**
		BDD100K	4980	0.932	0.884	0.075	0.062	0.038		**0.952**	**0.945**	**0.035**	**0.028**	**0.017**
		KITTI	5212	**0.975**	0.925	0.042	0.031	0.029		0.971	**0.942**	**0.015**	**0.012**	**0.015**
	Medium	nuScenes	21,763	0.898	0.842	0.096	0.085	0.042		**0.918**	**0.906**	**0.048**	**0.042**	**0.019**
		BDD100K	4980	0.915	0.865	0.082	0.074	0.038		**0.936**	**0.928**	**0.039**	**0.035**	**0.017**
		KITTI	5212	0.952	0.908	0.051	0.042	0.029		**0.968**	**0.960**	**0.021**	**0.018**	**0.015**
	Small	nuScenes	21,763	0.882	0.821	0.105	0.098	0.042		**0.902**	**0.891**	**0.055**	**0.051**	**0.019**
		BDD100K	4980	0.901	0.845	**0.045**	0.085	0.038		**0.921**	**0.912**	0.046	**0.042**	**0.017**
		KITTI	5212	0.938	0.889	**0.062**	0.055	0.029		**0.955**	**0.945**	0.078	**0.025**	**0.015**
CAPatch	Large	nuScenes	21,763	0.902	0.851	0.092	0.078	0.043		**0.922**	**0.910**	**0.045**	**0.039**	**0.019**
		BDD100K	4980	0.921	0.872	0.081	0.068	0.039		**0.941**	**0.932**	**0.038**	**0.032**	**0.018**
		KITTI	5212	**0.965**	0.912	0.048	0.035	0.030		0.955	**0.922**	**0.018**	**0.015**	**0.016**
	Medium	nuScenes	21,763	0.885	0.832	0.101	0.089	0.043		**0.905**	**0.894**	**0.051**	**0.046**	**0.019**
		BDD100K	4980	**0.925**	**0.915**	0.088	**0.069**	0.039		0.905	0.854	**0.072**	0.078	**0.018**
		KITTI	5212	0.941	0.895	0.055	0.048	0.030		**0.958**	**0.950**	**0.024**	**0.021**	**0.016**
	Small	nuScenes	21,763	0.868	0.805	0.112	0.105	0.043		**0.885**	**0.878**	**0.058**	**0.055**	**0.019**
		BDD100K	4980	0.888	0.828	0.098	0.092	0.039		**0.908**	**0.898**	**0.049**	**0.046**	**0.018**
		KITTI	5212	0.925	0.872	0.068	0.061	0.030		**0.942**	**0.935**	**0.031**	**0.028**	**0.016**
SLAP	Large	nuScenes	21,763	0.865	0.792	0.118	0.105	0.041		**0.892**	**0.876**	**0.055**	**0.048**	**0.019**
		BDD100K	4980	0.882	0.818	0.105	0.095	0.037		**0.912**	**0.894**	**0.048**	**0.042**	**0.017**
		KITTI	5212	0.925	0.872	0.065	0.055	0.028		**0.945**	**0.935**	**0.026**	**0.022**	**0.015**
	Medium	nuScenes	21,763	0.842	0.762	0.128	0.122	0.041		**0.875**	**0.852**	**0.065**	**0.058**	**0.019**
		BDD100K	4980	0.865	0.788	0.115	0.110	0.037		**0.895**	**0.878**	**0.055**	**0.048**	**0.017**
		KITTI	5212	0.905	0.852	0.075	0.068	0.028		**0.928**	**0.918**	**0.032**	**0.028**	**0.015**
	Small	nuScenes	21,763	0.818	0.728	0.145	0.142	0.041		**0.855**	**0.835**	**0.075**	**0.068**	**0.019**
		BDD100K	4980	**0.838**	**0.762**	0.132	0.125	0.037		0.825	0.758	**0.125**	**0.110**	**0.017**
		KITTI	5212	0.882	0.825	**0.082**	0.055	0.028		**0.912**	**0.895**	0.090	**0.035**	**0.015**

**Table 3:** Comparison with baselines on nuScenes under RP2 attack with large patches.

Method	DA	CA	FPR	FNR
DiffPure	0.546	0.271	0.365	0.241
PatchGuard	0.612	0.338	0.219	0.208
DetectorGuard	0.741	0.612	0.184	0.162
PercepGuard	0.863	0.731	0.236	0.151
PhySense	0.915	0.865	0.088	0.071
**Ours**	**0.935**	**0.921**	**0.041**	**0.035**

**Table 4:** Comprehensive black-box transferability analysis. The defense module is trained on **YOLOv8 (BDD100K)** and evaluated on unseen detectors (Faster R-CNN, CenterNet), datasets (nuScenes, KITTI), and attack types (RP2, CAPatch, SLAP) using **medium** patches. **Bold** indicates the source model performance.

Attack	Dataset	YOLOv8 (Source)					Faster R-CNN (Transfer)					CenterNet (Transfer)			
		DA	CA	FPR	FNR		DA	CA	FPR	FNR		DA	CA	FPR	FNR
RP2	nuScenes	**0.918**	**0.906**	**0.048**	**0.042**		0.826	0.770	0.098	0.092		0.815	0.758	0.105	0.099
	BDD100K	**0.936**	**0.928**	**0.039**	**0.035**		0.842	0.789	0.089	0.085		0.832	0.775	0.095	0.092
	KITTI	**0.968**	**0.960**	**0.021**	**0.018**		0.871	0.816	0.071	0.068		0.860	0.805	0.078	0.075
CAPatch	nuScenes	**0.905**	**0.894**	**0.051**	**0.046**		0.815	0.760	0.101	0.093		0.805	0.748	0.108	0.102
	BDD100K	**0.905**	**0.854**	**0.072**	**0.078**		0.815	0.726	0.122	0.121		0.802	0.715	0.128	0.135
	KITTI	**0.958**	**0.950**	**0.024**	**0.021**		0.862	0.808	0.074	0.071		0.850	0.795	0.082	0.078
SLAP	nuScenes	**0.875**	**0.852**	**0.065**	**0.058**		0.788	0.729	0.115	0.101		0.774	0.713	0.122	0.115
	BDD100K	**0.895**	**0.878**	**0.055**	**0.048**		0.806	0.746	0.105	0.091		0.795	0.732	0.113	0.105
	KITTI	**0.928**	**0.918**	**0.032**	**0.028**		0.835	0.780	0.082	0.078		0.825	0.765	0.089	0.085

**Table 5:** Defense performance of the proposed method under adaptive attackers on nuScenes.

Attack Setting	DA	CA	FPR	FNR
Non-Adaptive RP2	**0.935**	**0.921**	**0.041**	**0.035**
Trajectory-Smoothing RP2	0.805	0.786	0.118	0.115
TAME-Aware Joint Optimization	0.761	0.724	0.125	0.169
Frequency-Suppression Attack	0.784	0.725	0.121	0.136

**Table 6:** Ablation study on architectural coupling mechanisms on nuScenes.

Architecture Variant	RP2					SLAP			
	DA	CA	FPR	FNR		DA	CA	FPR	FNR
**Loose Coupling**	0.790	0.772	0.190	0.180		0.758	0.642	0.200	0.195
**Single Q**	0.810	0.795	0.165	0.160		0.775	0.761	0.180	0.175
**w/o Discrepancy**	0.820	0.708	0.170	0.146		0.780	0.768	0.182	0.169
**w/o Self-Attn**	0.800	0.785	0.155	0.181		0.760	0.748	0.170	0.203
**Full Arch (Ours)**	**0.935**	**0.921**	**0.041**	**0.035**		**0.895**	**0.878**	**0.055**	**0.048**

**Table 7:** Ablation on kinematic embedding strategies on nuScenes.

Embedding Variant	RP2					SLAP			
	DA	CA	FPR	FNR		DA	CA	FPR	FNR
**No Fourier**	0.813	0.800	0.260	0.155		0.770	0.753	0.278	0.170
**w/o High Freq**	0.785	0.770	0.230	0.110		0.760	0.745	0.235	0.115
**w/o Low Freq**	0.818	0.805	0.215	0.132		0.786	0.772	0.225	0.145
**Full Freq (Ours)**	**0.935**	**0.921**	**0.041**	**0.035**		**0.895**	**0.878**	**0.055**	**0.048**

**Table 8:** Ablation on TAME energy formulation and objectives.

Configuration	RP2					SLAP			
	DA	CA	FPR	FNR		DA	CA	FPR	FNR
**w/o Con-Reg** ($\lambda_{\mathrm{con}}=0$)	0.812	0.800	0.285	0.162		0.753	0.716	0.214	0.273
**w/o Adv-Calib** ($\lambda_{\mathrm{adv}}=0$)	0.798	0.784	0.250	0.192		0.744	0.732	0.252	0.205
**Asym. KL**	0.822	0.810	0.226	0.150		0.686	0.671	0.288	0.193
**L1 Distance**	0.710	0.698	0.255	0.170		0.680	0.668	0.268	0.182
**Full TAME (Ours)**	**0.935**	**0.921**	**0.041**	**0.035**		**0.895**	**0.878**	**0.055**	**0.048**

## Data Availability

You can find the dataset we use in this experiment at https://www.cvlibs.net/datasets/kitti (KITTI), https://www.nuscenes.org/nuscenes##data-collection (nuScenes), http://bdd-data.berkeley.edu/download.html (BDD100K), accessed on 8 December 2025. The codes supporting the result of this study will be made available by the author on request.
